# Deletion of *Caldicellulosiruptor bescii* CelA reveals its crucial role in the deconstruction of lignocellulosic biomass

**DOI:** 10.1186/s13068-014-0142-6

**Published:** 2014-10-09

**Authors:** Jenna Young, Daehwan Chung, Yannick J Bomble, Michael E Himmel, Janet Westpheling

**Affiliations:** Department of Genetics, University of Georgia, Athens, Georgia; Biosciences Center, National Renewable Energy Laboratory, Golden, CO USA; The BioEnergy Science Center, Oak Ridge National Laboratory, Oak Ridge, TN USA

**Keywords:** Bioenergy, Cellulase, Thermophile

## Abstract

**Background:**

Members of the bacterial genus *Caldicellulosiruptor* are the most thermophilic cellulolytic organisms described to date, and have the ability to grow on lignocellulosic biomass without conventional pretreatment. Different species vary in their abilities to degrade cellulose, and the presence of CelA, a bifunctional glycoside hydrolase that contains a Family 48 and a Family 9 catalytic domain, correlates well with cellulolytic ability in members of this genus. For example, *C. hydrothermalis*, which does not contain a CelA homolog, or a GH48 Family or GH9 Family glycoside hydrolase, is the least cellulolytic of the *Caldicellulosiruptor* species so far described. *C. bescii*, which contains CelA and expresses it constitutively, is among the most cellulolytic. In fact, CelA is the most abundant extracellular protein produced in *C. bescii*. The enzyme contains two catalytic units, a Family 9A-CBM3c processive endoglucanase and a Family 48 exoglucanase, joined by two Family 3b carbohydrate-binding domains. Although there are two non-reducing end-specific Family 9 and three reducing end-specific Family 48 glycoside hydrolases (producing primarily glucose and cellobiose; and cellobiose and cellotriose, respectively) in *C. bescii*, CelA is the only protein that combines both enzymatic activities.

**Results:**

A deletion of the *celA* gene resulted in a dramatic reduction in the microorganism’s ability to grow on crystalline cellulose (Avicel) and diminished growth on lignocellulosic biomass. A comparison of the overall endoglucanase and exoglucanase activities of the mutant compared with the wild-type suggests that the loss of the endoglucanase activity provided by the GH9 family domain is perhaps compensated for by other enzymes produced by the cell. In contrast, it appears that no other enzymes in the *C. bescii* secretome can compensate for the loss of exoglucanase activity. The change in enzymatic activity in the *celA* mutant resulted in a 15-fold decrease in sugar release on Avicel compared with the parent and wild-type strains.

**Conclusions:**

The exoglucanase activity of the GH48 domain of CelA plays a major role in biomass degradation within the suite of *C. bescii* biomass-degrading enzymes.

**Electronic supplementary material:**

The online version of this article (doi:10.1186/s13068-014-0142-6) contains supplementary material, which is available to authorized users.

## Background

The native recalcitrance of lignocellulosic biomass remains the major barrier to the conversion of these substrates to biofuels [1-3]. Conversion of biomass typically involves pretreatment of the biomass with acid or base at high temperature, followed by enzymatic hydrolysis before fermentation of the released sugars to fuels, such as ethanol. Consolidated bioprocessing (CBP) allows the combination of enzymatic hydrolysis and sugar conversion in one step, and therefore reduces costs [4]. Thermophilic CBP organisms are of particular interest, because industrial processing at high temperatures increases enzymatic rates, reduces the risk of contamination, and allows more efficient separation/purification of ethanol [5]. Organisms from the hyperthermophilic genus *Caldicellulosiruptor* are of particular interest, as some members have the ability to utilize biomass without the need for conventional pretreatment [6,7]. For example, we have recently demonstrated that engineered *Caldicellulosiruptor bescii* converts untreated switchgrass directly to ethanol [8].

*C. bescii* produces a suite of secreted enzymes, including 52 glycoside hydrolases, which allows it to break down the carbohydrate components of plant cell walls [9]. These enzymes include glycoside hydrolases and carbohydrate esterases linked to carbohydrate-binding modules [9,10]. Of particular interest are the multifunctional proteins that have more than one catalytic domain linked to carbohydrate-binding domains [10]. One such cellulase, CelA, is the most abundant protein secreted by *C. bescii* [7,11] and has been shown to outperform mixtures of commercially available exoglucanases and endoglucanases *in vitro* [12]. CelA consists of a Family 9A-CBM3c processive endoglucanase and a Family 48 exoglucanase (two GH families that are known to be synergistic [13]), connected by a linker region containing two Family 3b carbohydrate-binding domains. This combination creates a dual mode of action on cellulose, in which the processive endoglucanase breaks internal cellulose bonds, creating new chain ends for the exoglucanase [10,14,15]. These multifunctional combinations of cellulolytic enzymatic activity in one protein (i.e. gene product) are fundamentally distinct from the multi-domain cellulosomes observed in other cellulolytic anaerobes, such as *Clostridium thermocellum* [16-18]. Cellulosomes are highly complexed protein aggregates (up to nine catalytic domains per scaffold) that are also self-assembling [7,11,12]. Unlike cellulosomes, most *Caldicellulosiruptor* enzymes exist as free enzymes that do not remain associated with the cell. Recent work suggests that CelA acts by both conventional cellulase processivity and excavation of cavities into the surface of the biomass substrate [12].

Whereas *C. bescii* encodes many cellulolytic enzymes, some of which are induced by growth on biomass substrates, CelA RNA is abundant throughout growth and is thus apparently not dependent on induction by biomass carbon sources [19]. A deletion of the gene encoding CelA in *C. bescii* was constructed to assess its role in biomass deconstruction in the context of other cellulolytic biomass-degrading enzymes *in vivo*. Growth of the mutant was unaffected on the soluble substrate, cellobiose, but was reduced on the insoluble substrates: 38% on *Populus trichocarpa* (poplar), 20% on *Panicum virgatum* L. (switchgrass), 27% on *Arabidopsis thaliana*, and 77% on Avicel. Interestingly, the growth defect was more pronounced on Avicel (a model cellulose) than on lignocellulosic biomass. Analysis of the extracellular enzymatic activity of the mutant compared with the parent strain showed that the mutant was severely reduced in exoglucanase activity as measured by hydrolysis of Avicel, but not endoglucanase activity as measured by hydrolysis of carboxymethylcellulose (CMC).

## Results and discussion

### Deletion of *celA* results in reduced growth on the insoluble substrates *Populus trichocarpa* (poplar), *Panicum virgatum* (switchgrass), *Arabidopsis thaliana*, and Avicel, but not the soluble substrate, cellobiose

**Figure 1:**
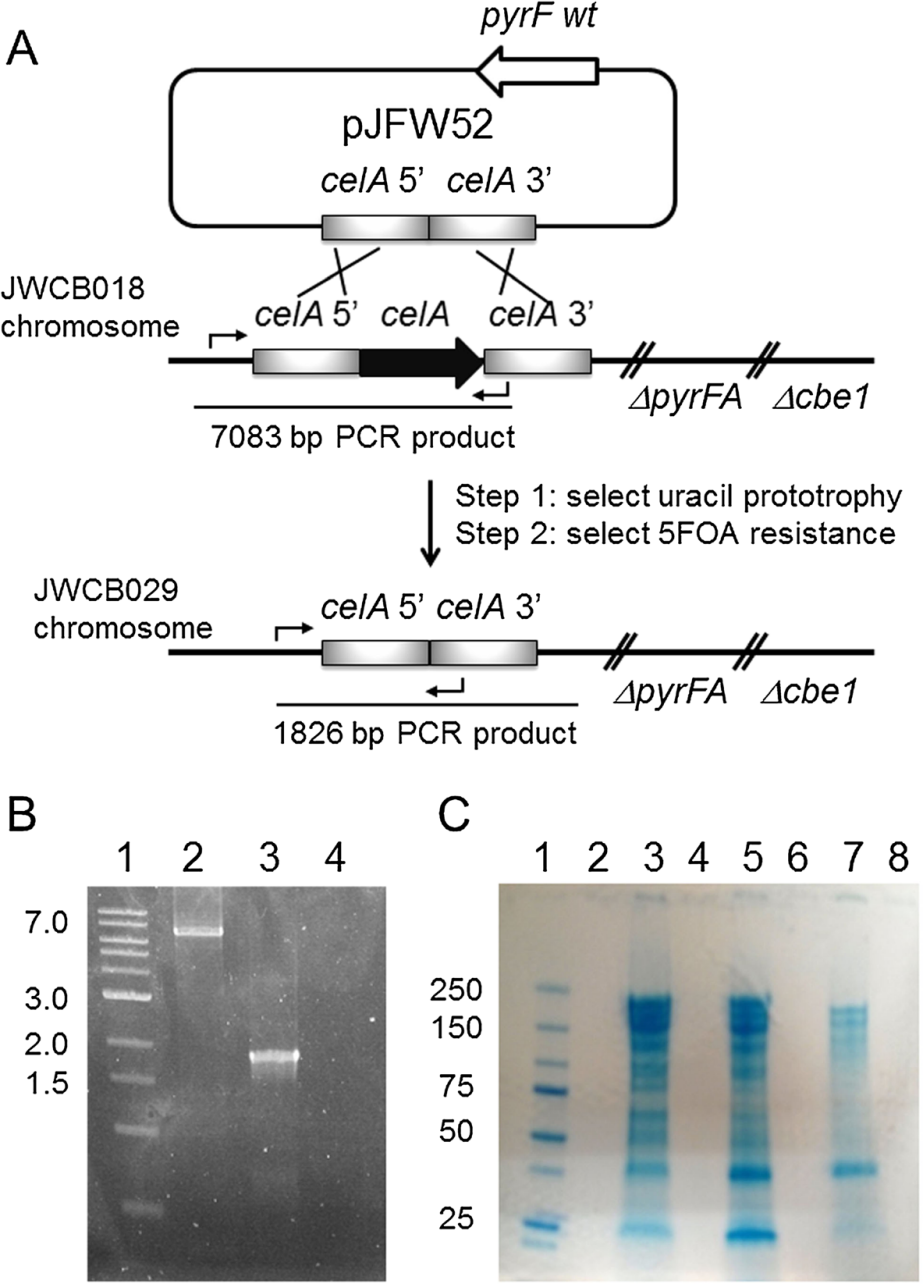
**Deletion of the** ***celA*** **gene in** ***Caldicellulosiruptor bescii*** **. (A)** Depiction of the chromosomal location of *celA* and construction of the gene deletion. A deletion cassette was constructed in a non-replicating plasmid, pJFW52, which contained a wild-type copy of the *pyrF* gene for prototropic selection of transformants in a strain containing a *pyrFA* deletion. The cassette contained *celA* 5′ and 3′ flanking DNA fragments. The plasmid was transformed into JWCB018 (*ΔpyrFA ΔcbeI*), and uracil prototrophs were selected (resulting from plasmid insertion). Counter-selection with 5-FOA selected for strains that underwent a second recombination event, some of which resulted in deletion of *celA* to produce strain JWCB029 (*ΔpyrFA ΔcbeI ΔcelA*). **(B)** Agarose gel showing PCR products amplified using primers JF200 and DC432 from the *celA* locus in the parent strain JWCB018 (lane 2) and the the *celA* deletion strain, JWCB029 (lane 3). Lane 1: DNA MW standards; lane 4: no template PCR control. Expected bands: wild-type *celA* locus: 7.1 kb; *celA* deletion: 1.8 kb. **(C)** SDS-PAGE gel stained with Coomassie Brilliant Blue showing precipitated extracellular protein from the wild-type JWCB001 (lane 3), JWCB018 (lane 5), and JWCB029 (lane 7), standards (Precision Plus Protein™ Dual Color Standards; BioRad) in lane 1.

The region of the *C. bescii* chromosome containing the *celA* gene is depicted in Figure 1A. A vector for targeted deletion of *celA*, pJFW52 (Additional file 1: Figure S1), was constructed by joining 1 kb of the upstream and 1 kb of the downstream region of the CelA (Cbes1867) open reading frame, deleting the open reading frame itself. The plasmid also contained a wild-type allele of the *pyrF* gene, but no origin of replication for *C. bescii*. pJFW52 was used to transform *C. bescii* JWCB018, which contains a deletion of the *pyrFA* locus that results in uracil auxotrophy. Plasmid transformants were selected for uracil prototrophy, resulting in plasmid integration at the *celA* locus. Counter-selection with 5-fluoroorotic acid (5-FOA) which is converted to a toxic product by the wild-type *pyrF* allele, was used to select recombinants that had lost the wild-type allele (5-FOA resistance) by plasmid excision, and those were screened for deletions of *celA* (Figure 1A). The JWCB018 strain also contains a deletion of *cbeI*, an endonuclease that inhibits DNA transformation [20,21]. Deletion of *celA* was confirmed by PCR amplification of the gene region using primers upstream and downstream of Cbes1867. The deletion resulted in a 1.83 kb fragment compared with the wild-type fragment of 7.08 kb (Figure 1B). Primers that annealed within the *celA* gene produced the predicted product from the wild-type strains, but failed to produce a product in the deletion strain (data not shown). Extracellular proteins from the mutant (JWCB029), the wild-type (JWCB001), and the parent strain (JWCB018) were concentrated and displayed by SDS-PAGE stained with Coomassie Brilliant Blue. As shown in Figure 1C, a protein of the predicted size of CelA (approximately 230 kDa [14]), was shown to be present in the wild-type and parent strains, but was absent in the *celA* deletion strain.

**Figure 2:**
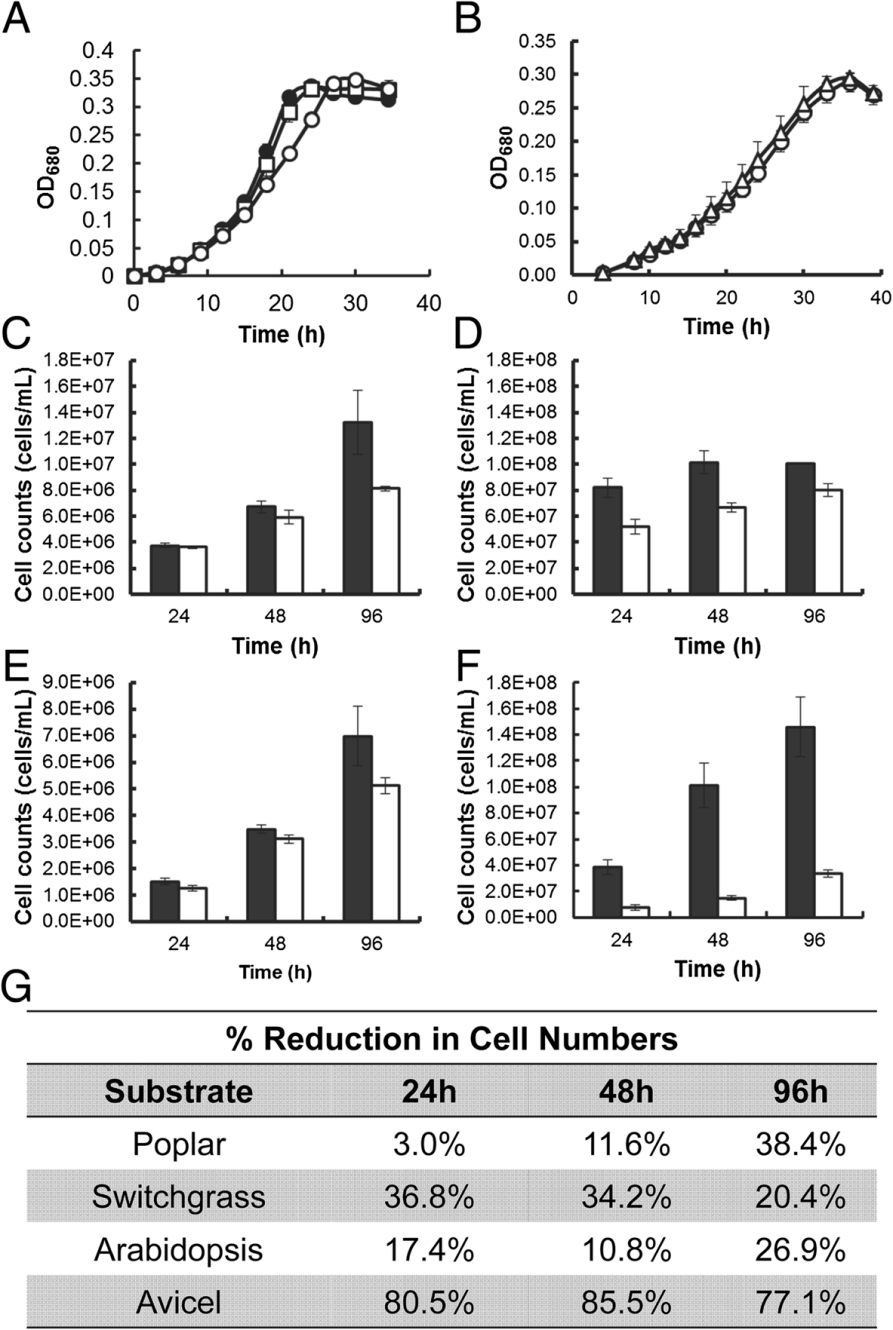
**Growth of the wild-type, JWCB005 (** ***ΔpyrFA*** **), JWCB018 (** ***ΔpyrFA ΔcbeI*** **), and JWCB029 (** ***ΔpyrFA ΔcbeI ΔcelA*** **) strains on various carbon sources. (A, B)** growth of the wild-type (closed circles), JWCB005 (open squares), JWCB018 (open circles), and JWCB029 (open triangles) strains on cellobiose. **(C-F)** Growth of the JWCB018 (solid bars), and JWCB029 (open bars) strains on **(C)** *Populus trichocarpa (*poplar), **(D)** *Panicum virgatum* L. (switchgrass), **(E)** *Arabidopsis thaliana*, and **(F)** Avicel. **(G)** Growth as a percentage reduction in cell numbers of the CelA deletion strain, JWCB029, compared to the background strain JWCB018.

To examine the phenotype of the *celA* deletion mutant, growth was first measured on the soluble substrate, cellobiose. Growth of the wild-type, parent, JWCB018 [21-23] (*ΔpyrFA ΔcbeI*), and JWCB005 [23] (*ΔpyrFA*) strains were virtually indistinguishable from the mutant, JWCB029 (*ΔpyrFA ΔcbeI ΔcelA*) (Figure 2A, B), suggesting that the *celA* mutant elicited no general growth defect. To determine growth on insoluble substrates, cells were stained with Acridine Orange and counted using fluorescence microscopy. We note that in our experience, growth on complex biomass as measured by optical density is not reliable, because it is difficult to distinguish cells from substrate particles.

After 96 hours of incubation on *Populus trichocarpa* (poplar), *Panicum virgatum* (switchgrass), *Arabidopsis thaliana*, and Avicel, growth yields were diminished by 38%, 20%, 27%, and 77% respectively, compared with the parental strain (JWCB018) (Figure 2C-G), indicating that CelA plays a crucial role in complex biomass utilization.

Interestingly, this difference in growth on lignocellulose was similar to that seen in a Cel48S deletion strain of *C. thermocellum* [24]. Like CelA in *C. bescii*, Cel48S is the most abundant cellulase in *C. thermocellum*. Lignocellulosic biomass is a complex substrate with a variety of glycosidic bonds. CelA is the most abundant extracellular protein produced by *C. bescii* and is apparently produced constitutively. Two other genes reported to be upregulated during growth on biomass, Cbes1857 (upregulated four-fold), which contains a GH48 family domain, and Cbes1865 (upregulated 23-fold) [19], which contains a GH9 family domain, might partially compensate for the loss of CelA during growth on lignocellulosic biomass. Additionally, *C. bescii* is able to grow and utilize xylan as sole carbon source [7], and given that the biomass used in this study contains close to 20% xylan (Additional file 1: Table S2), *C. bescii* may therefore use xylan primarily for initial growth. The use of xylose may also allow *C. bescii* to produce enough cellulases in the CelA mutant to degrade cellulose, the more recalcitrant cell wall polysaccharide.

**Figure 3:**
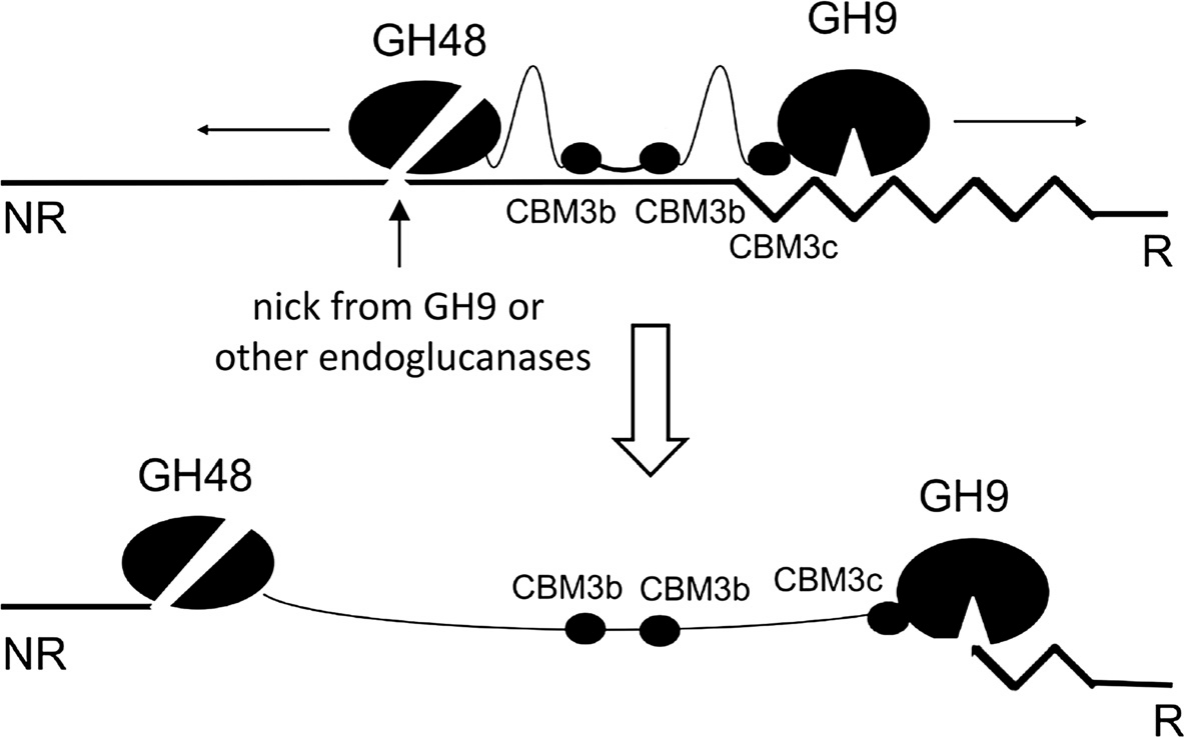
**Schematic depiction of the putative behavior of CelA on cellulose.** The GH48 catalytic domain primarily degrades crystalline cellulose (straight line); whereas the GH9 degrades amorphous regions (jagged line) and processively creates nicks in the crystalline regions for the GH48 catalytic domain to engage. As long as the CBMs are actively bound to the substrate, CelA will produce cavities because the length of the linker peptide limits the separation of the catalytic domains.

The most dramatic phenotype of the *C. bescii celA* mutant was observed during growth on Avicel, which showed a 77% reduction in growth. Commercial Avicel is a model cellulosic substrate used for enzymatic hydrolysis and is known to contain about 33% amorphous cellulose and 67% crystalline cellulose [25]. It is produced by acid hydrolysis of cellulosic substrates, which removes almost all hemicellulose [26]. CelA is one of 52 glycoside hydrolases secreted by this strain that are potentially capable of digesting Avicel, but is the only enzyme that combines GH9 and GH48 activities in the same polypeptide. The combination of a Family 9A-CBM3c processive endoglucanase and a Family 48 exoglucanase connected by a linker region with two Family 3b CBMs provides a synergistic mode of action that makes the activity of CelA unique [13]. This natural chimeric construct creates a hyperactive cellulase in which the endoglucanase breaks internal cellulose bonds, creating chain ends for the processive exoglucanase [10,14,15]. In fact, it was recently shown that CelA degrades Avicel using a novel digestion mechanism by which CelA creates cavities within the substrate, along with the more common ablative mechanism used by most fungal and bacterial exoglucanases [12]. The combination of a reducing end-specific exoglucanase (GH48) and a non-reducing end-specific processive endoglucanase (GH9-CBM3c) connected by Family 3b CBMs is most likely responsible for this unique mechanism (Figure 3). This mechanism not only promotes fast hydrolysis by CelA, but can also benefit other less efficient cellulases produced by *C. bescii*, as it greatly increases the accessible surface area of the substrate that is available for hydrolysis. CelA is the only cellulase in *C. bescii* that combines these two complementary catalytic domains into one gene product. From these results, it appears that no other cellulase displays this combined activity, or that the separate activities are not sufficiently upregulated to compensate for the absence of CelA.

### Reduced cellulase activity in the *celA* deletion mutant results from loss of exoglucanase activity

**Figure 4:**
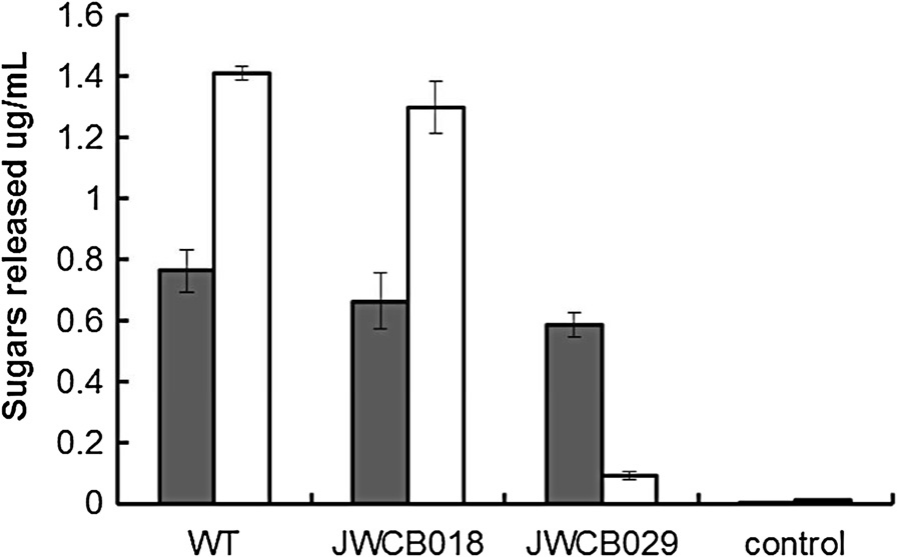
**Cellulolytic activity (reducing sugars released by** ***in vitro*** **extracellular protein) of the extracellular fraction of** ***Caldicellulosiruptor bescii*** **grown on cellobiose, on carboxymethylcellulose (CMC) (gray bars) after 1 hour and Avicel (white bars) after 24 hours incubation with the extracellular fraction of wild-type, JWCB018 (** ***ΔpyrFA ΔcbeI*** **), and JWCB029 (** ***ΔpyrFA ΔcbeI ΔcelA*** **) strains.**

CMC is a soluble form of cellulose specifically used for estimating endoglucanase activity. Avicel, a microcrystalline form of cellulose, is primarily used to estimate exoglucanase activity. Because CelA is a bifunctional enzyme with both endoglucanase and exoglucanase activity, both substrates were used to assay cellulolytic activity in the *celA* mutant. Extracellular proteins from the control cultures and the *celA* deletion strain grown on cellobiose were tested for cellulolytic activity. The activity of the wild-type and parent strain resulted in comparable sugar release, ranging from around 0.65 to 0.75 μg/ml sugar released from CMC, and 1.3 to 1.4 μg/ml sugar released on Avicel (Figure 4). The *celA* deletion strain showed a similar profile on CMC (0.6 μg/ml sugar released) to that of the wild-type and parent strains; however, a dramatic reduction in enzyme activity was observed on Avicel (approximately 0.1 μg/ml sugar released). The presence of endoglucanase activity in the mutant suggests that this activity, attributed to the GH9 family domain of CelA, is redundant in the genome and may partially compensate for the loss of CelA [27]. A recent study showed that the endoglucanase activity of the GH9 domain provides substrate for the exoglucanase activity of the GH48 domain [15], suggesting that this is the rate-limiting activity for this enzyme. The apparent need for an abundant amount of endoglucanase activity may explain the redundancy of these genes in the genome and the upregulation of their RNA transcripts during growth on biomass [19]. Interestingly, the Family 9 glycoside hydrolase of *Clostridium phytofermentans* was determined to be essential for growth on filter paper [27]. *C. phytofermentans* contains twice as many (108) glycosyl hydrolases as *C. bescii*, but contains only one GH9 family endoglucanase [27]. If the activity of the GH9 enzyme is rate-limiting, the redundancy of these enzymes in *C. bescii* may ensure enough GH9 non-reducing end-specific digestion of the substrate to allow reducing end-specific enzymes to deconstruct the biomass.

In contrast, the enzymatic release of sugars on Avicel by the mutant was reduced approximately 15-fold compared to the parent and wild-type strains (Figure 4), suggesting that the exoglucanase activity of the CelA GH48 domain plays a major role in cellulase degradation within the suite of enzymes in *C. bescii*, in spite of the presence of two other GH48 Family enzymes. These data suggest that the exoglucanase activity supplied by CelA may be the primary source of this activity in *C. bescii*. As shown in Figure 3, the power of the CelA system itself may arise from its ability to form cavities in cellulose by virtue of its specific tethered structure (linking a reducing chain end-specific exoglucanase to a non-reducing chain end-specific endoglucanase). In addition to this unique cellulose degrading strategy, CelA may also fragment during growth on biomass, presenting the more common free processive endoglucanase and exoglucanase activities to bear on cellulosic substrates, as suggested by the *C. bescii* secretome fractionation shown in Brunecky et al. [12].

## Conclusions

Diminished growth on insoluble lignocellulosic substrates in the *celA* mutant strain demonstrated the importance of this enzyme within the suite of enzymes secreted by *C. bescii* that contribute to its powerful cellulolytic capability. Reduced exoglucanase activity in the mutant suggests that the GH48 Family domain of CelA, in particular, provides the primary exoglucanase activity in the *C. bescii* enzyme cocktail.

## Methods

### Strains, media, and growth conditions

*Caldicellulosiruptor* and *Escherichia coli* strains used in this study are listed in Table 1. *Caldicellulosiruptor* strains were grown anaerobically at 75°C on solid or in liquid low osmolarity defined (LOD) medium [28], as previously described, with maltose, cellobiose, poplar, switchgrass, *Arabidopsis,* or Avicel as sole carbon source, as indicated. Uracil (40 μM) was added to the growth media to supplement nutritional mutants unless otherwise indicated. This concentration of uracil does not support growth of *C. bescii* as sole carbon source. *E. coli* strain DH5α was used for plasmid DNA construction and preparation using standard techniques [29]. *E. coli* cells were cultured in LB broth supplemented with apramycin (50 μg/ml), and plasmid DNA was isolated using a Qiagen (Hilden, Germany) Miniprep kit. Chromosomal DNA from *Caldicellulosiruptor* strains was extracted using the Quick-gDNA™ MiniPrep (Zymo, Irvine, CA) as previously described [21].

**Table 1:** **Strains/plasmid used in this study**

**Strains/plasmid**	**Genotype/phenotype**	**Source**
*Caldicellulosiruptor bescii* JWCB001	DSMZ6725 wild type (ura^+^/5-FOA^S^)	DSMZ^a^
*C. bescii* JWCB005	*ΔpyrFA* (ura^−^/5-FOA^R^)	[23]
*C. bescii* JWCB018	*ΔpyrFA ldh::ISCbe4 Δcbe1* (ura^−^/5-FOA^R^)	[21,22]
*C. bescii* JWCB029	*ΔpyrFA ldh::ISCbe4 Δcbe1 ΔcelA* (ura^−^/5-FOA^R^)	This study
*Escherichia coli* JW330	DH5α containing pJFW52 (Apramycin^R^)	This study
pJFW52	*celA* (Cbes1867) deletion vector (Apramycin^R^)	This study

^a^German Collection of Microorganisms and Cell cultures.

### Construction of a *celA* deletion in the *Caldicellulosiruptor bescii* chromosome

Using *C. bescii* (JWCB018) genomic DNA as template, a 2,097 bp fragment containing the 5′ and 3′ flanking regions of *celA* (Cbes1867) was generated by overlap extension PCR (OE-PCR) using primers CelA-5, JF006, JF007, and CelA-3, with a *Kpn*I site added to the 5′ end and an *Apa*LI site at the 3′ end. A fragment containing an apramycin-resistance gene cassette, a *pyrF* cassette [23], and the *E. coli pSC101* replication origin, was amplified from pDCW89 [23] using primers DC081 and DC262 with the same restriction sites added. The two linear DNA fragments were digested with *Kpn*I and *Apa*LI, and ligated to generate pJFW52 (see Additional file 1: Figure S1). DNA sequences of the primers used in this construction are shown (see Additional file 1: Table S1). *E. coli* strain DH5α was transformed by electroporation in a 2-mm-gap cuvette at 2.5 V, and plasmid was isolated using a Qiagen Miniprep Kit. Electrotransformation of JWCB018 was performed as previously described [20]. Recovery cultures, electropulsed with plasmid DNA (approximately 0.5 μg), were transferred to defined minimal medium [28] without uracil to allow selection of uracil prototrophs. DNA was isolated from transformants, and PCR amplification, using primers upstream and downstream of the targeted deletion (JF200 and DC432), was used to confirm the presence of the deletion. Transformants were inoculated into nonselective liquid defined medium, with 20 μM uracil, and incubated overnight at 75°C. A set of serial dilutions of the overnight culture were plated directly onto defined medium containing 8 mM 5-FOA and 20 μM uracil as described [21]. Colonies resistant to 5-FOA were cultured in medium containing uracil for genomic DNA isolation and PCR screening of the targeted region. Primers (JF200 and DC432) designed to amplify upstream and downstream of the homologous regions were used to construct the deletion (see Additional file 1: Table S1). The PCR extension time was sufficient to allow amplification of the wild-type allele, if it was still present. After initial screening, transformants containing the expected deletion were further purified and screened by PCR to ensure segregation of the deleted allele. Another set of primers, one located inside the Cbes1867 open reading frame, and the other located outside the flanking region, were used for further verification. The PCR products were then sequenced to verify the site of the deletion.

### Growth of the *celA* deletion mutant on soluble and insoluble substrates

Cell growth was monitored on the soluble substrate, cellobiose, by optical density (680 nm) using a Jenway Genova spectrophotometer. The insoluble substrates used in this study were *P. trichocarpa* (poplar), *P. virgatum* L. (switchgrass), *A. thaliana*, and Avicel PH-101 (Sigma). For biomass composition of these substrates, see Additional file 1: Table S2. To monitor growth on these unwashed insoluble substrates (0.5% (w/v)), cultures were sampled and fixed in 3.7% formaldehyde, vortexed, and stored at −20°C for cell counts. Samples were appropriately diluted and stained with 0.1% Acridine Orange before visualizing using an epifluorescent microscope at 100× magnification (oil immersion). Cell counts from 15 to 20 fields were averaged.

### Cellulase enzyme activity assays

Cultures were transferred on cellobiose and then inoculated at 5% into two 400 ml bottles of LOD medium with cellobiose as sole carbon source. When growth reached an OD_680_ of approximately 0.3, the supernatant was harvested by centrifugation (6000 rpm, 2 × 30 min). Ammonium sulfate (80% saturation) was added to the supernatant slowly while mixing at 4°C and then allowed to mix overnight. Precipitated protein was recovered by centrifugation at 14,321 × *g* for 25 min and resuspended in 20 mM MES buffer with 2 mM β-mercaptoethanol (1 ml) [30]. Protein concentrations were determined using BioRad Protein Assay reagent with BSA as the standard, in accordance with the manufacturer’s instructions. Cellulolytic activity was determined using 10 g/L CMC or Avicel in MES reaction buffer (pH 5.5) as previously described [30]; 50 μg of precipitated extracellular protein was added to each reaction and incubated at 75°C (1 hour for CMC and 24 hours for Avicel). Controls were incubated for the same time without added enzyme. Reducing sugars in the supernatant were measured using dinitrosalyclic acid (DNS). Samples and standards (glucose) were mixed 1:1 with DNS, boiled for 5 min and measured at OD_575_. Activity was reported as μg/ml of sugar released.

### Protein gel electrophoresis

Precipitated supernatant protein (50 μg) was analyzed by SDS-PAGE using a 4 to 15% gradient gel (BioRad precast) run at 150 V for 1 hour. Proteins were visualized by staining with Coomassie Brilliant Blue.

## Additional file

Additional file 1: Figure S1. Diagram of the *celA* (Cbes1867) deletion vector. The white colored arrows indicate sequences originating from *Caldicellulosiruptor bescii* and sequences originating from *E. coli* are indicated as black arrows. The apramycin resistant gene cassette (Apr^R^); *pSC101,* low copy replication origin in *E. coli*; *repA*, a plasmid-encoded gene required for *pSC101* replication; *par*, partition locus; *pyrF* cassette; 5’ and 3’ flanking sequences of the *celA* (Cbes1867) site in *C. bescii* chromosome are indicated. All Primers and the two restriction sites (KpnI and ApaLI) used in this construction are also indicated. **Table S1.** Primers used in this study. **Table S2.** Approximate Biomass Composition of insoluble substrates.

## Abbreviations

CBP: Consolidated bioprocessing
CMC: Carboxymethylcellulose
DNS: Dinitrosalicylic acid
GH: Glycoside hydrolase
LOD: Low osmolarity defined
SDS-PAGE: Sodium dodecyl sulfate-polyacrylamide gel electrophoresis

## References

[1] Himmel ME, Ding SY, Johnson DK, Adney WS, Nimlos MR, Brady JW, Foust TD (2007). Biomass recalcitrance: Engineering plants and enzymes for biofuels production. Science.

[2] McCann MC, Carpita NC (2008). Designing the deconstruction of plant cell walls. Curr Opin Plant Biol.

[3] Wilson DB (2008). Three microbial strategies for plant cell wall degradation. Ann Ny Acad Sci.

[4] Lynd LR, van Zyl WH, McBride JE, Laser M (2005). Consolidated bioprocessing of cellulosic biomass: an update. Curr Opin Biotechnol.

[5] Lin L, Xu J (2013). Dissecting and engineering metabolic and regulatory networks of thermophilic bacteria for biofuel production. Biotechnol Adv.

[6] Blumer-Schuette SE, Kataeva I, Westpheling J, Adams MWW, Kelly RM (2008). Extremely thermophilic microorganisms for biomass conversion: status and prospects. Curr Opin Biotechnol.

[7] Yang SJ, Kataeva I, Hamilton-Brehm SD, Engle NL, Tschaplinski TJ, Doeppke C, Davis M, Westpheling J, Adams MWW (2009). Efficient degradation of lignocellulosic plant biomass, without pretreatment, by the thermophilic anaerobe "*Anaerocellum thermophilum*" DSM 6725. Appl Environ Microbiol.

[8] Chung DCM, Guss A, Westpheling J (2014). Direct conversion of plant biomass to ethanol by *Caldicellulosiruptor bescii*. Proc Natl Acad Sci U S A.

[9] Blumer-Schuette SE, Giannone RJ, Zurawski JV, Ozdemir I, Ma Q, Yin YB, Xu Y, Kataeva I, Poole FL, Adams MWW, Hamilton-Brehm SD, Elkins JG, Larimer FW, Land ML, Hauser LJ, Cottingham RW, Hettich RL, Kelly RM (2012). *Caldicellulosiruptor* Core and pangenomes reveal determinants for noncellulosomal thermophilic deconstruction of plant biomass. J Bacteriol.

[10] Dam P, Kataeva I, Yang SJ, Zhou FF, Yin YB, Chou WC, Poole FL, Westpheling J, Hettich R, Giannone R, Lewis DL, Kelly R, Gilbert HJ, Henrissat B, Xu Y, Adams MWW (2011). Insights into plant biomass conversion from the genome of the anaerobic thermophilic bacterium *Caldicellulosiruptor bescii* DSM 6725. Nucleic Acids Res.

[11] Lochner A, Giannone RJ, Rodriguez M, Shah MB, Mielenz JR, Keller M, Antranikian G, Graham DE, Hettich RL (2011). Use of label-free quantitative proteomics to distinguish the secreted cellulolytic systems of *Caldicellulosiruptor bescii* and *Caldicellulosiruptor obsidiansis*. Appl Environ Microbiol.

[12] Brunecky R, Alahuhta M, Xu Q, Donohoe BS, Crowley MF, Kataeva IA, Yang SJ, Resch MG, Adams MW, Lunin VV, Himmel ME, Bomble YJ (2013). Revealing nature's cellulase diversity: the digestion mechanism of *Caldicellulosiruptor bescii* CelA. Science.

[13] Vazana Y, Morais S, Barak Y, Lamed R, Bayer EA (2010). Interplay between *Clostridium thermocellum* family 48 and family 9 cellulases in cellulosomal versus noncellulosomal states. Appl Environ Microbiol.

[14] Zverlov V, Mahr S, Riedel K, Bronnenmeier K (1998). Properties and gene structure of a bifunctional cellulolytic enzyme (CelA) from the extreme thermophile '*Anaerocellum thermophilum*' with separate glycosyl hydrolase family 9 and 48 catalytic domains. Microbiology.

[15] Yi Z, Su X, Revindran V, Mackie RI, Cann I: **Molecular and biochemical analyses of CbCel9A/Cel48A, a highly secreted multi-modular cellulase, by*****Caldicellulosiruptor bescii*****during growth on crystalline cellulose.** *PLoS One* 2013, **8:**e84172. 10.1371/journal.pone.0084172 PMC3865294 24358340

[16] Gilbert HJ (2007). Cellulosomes: microbial nanomachines that display plasticity in quaternary structure. Mol Microbiol.

[17] Bayer EA, Morag E, Lamed R (1994). The Cellulosome - a treasuretrove for biotechnology. Trends Biotechnol.

[18] Bayer EA, Setter E, Lamed R (1985). Organization and distribution of the cellulosome in *Clostridium thermocellum*. J Bacteriol.

[19] Kataeva I, Foston MB, Yang SJ, Pattathil S, Biswal AK, Poole FL, Basen M, Rhaesa AM, Thomas TP, Azadi P, Olman V, Saffold TD, Mohler KE, Lewis DL, Doeppke C, Zeng YN, Tschaplinski TJ, York WS, Davis M, Mohnen D, Xu Y, Ragauskas AJ, Ding SY, Kelly RM, Hahn MG, Adams MWW (2013). Carbohydrate and lignin are simultaneously solubilized from unpretreated switchgrass by microbial action at high temperature. Energ Environ Sci.

[20] Chung D, Farkas J, Huddleston JR, Olivar E, Westpheling J: **Methylation by a unique alpha-class N4-cytosine methyltransferase is required for DNA transformation of*****Caldicellulosiruptor bescii*****DSM6725.** *PLoS One* 2012, **7:**e43844. 10.1371/journal.pone.0043844 PMC3425538 22928042

[21] Chung DH, Huddleston JR, Farkas J, Westpheling J (2011). Identification and characterization of CbeI, a novel thermostable restriction enzyme from *Caldicellulosiruptor bescii* DSM 6725 and a member of a new subfamily of HaeIII-like enzymes. J Ind Microbiol Biotechnol.

[22] Cha M, Wang H, Chung D, Bennetzen JL, Westpheling J (2013). Isolation and bioinformatic analysis of a novel transposable element, ISCbe4, from the hyperthermophilic bacterium, *Caldicellulosiruptor bescii*. J Ind Microbiol Biotechnol.

[23] Chung D, Cha M, Farkas J, Westpheling J: **Construction of a stable replicating shuttle vector for*****Caldicellulosiruptor*****species: use for extending genetic methodologies to other members of this genus.** *PLoS One* 2013, **8:**e62881. 10.1371/journal.pone.0062881 PMC3643907 23658781

[24] Olson DG, Tripathi SA, Giannone RJ, Lo J, Caiazza NC, Hogsett DA, Hettich RL, Guss AM, Dubrovsky G, Lynd LR (2010). Deletion of the Cel48S cellulase from *Clostridium thermocellum*. Proc Natl Acad Sci U S A.

[25] Park S, Baker JO, Himmel ME, Parilla PA, Johnson DK: **Cellulose crystallinity index: measurement techniques and their impact on interpreting cellulase performance.** *Biotechnology Biofuels* 2010, **3:**10. 10.1186/1754-6834-3-10 PMC2890632 20497524

[26] Gao S, You C, Renneckar S, Bao J, Zhang YH: **New insights into enzymatic hydrolysis of heterogeneous cellulose by using carbohydrate-binding module 3 containing GFP and carbohydrate-binding module 17 containing CFP.** *Biotechnology Biofuels* 2014, **7:**24. 10.1186/1754-6834-7-24 PMC3943381 24552554

[27] Tolonen AC, Chilaka AC, Church GM (2009). Targeted gene inactivation in *Clostridium phytofermentans* shows that cellulose degradation requires the family 9 hydrolase Cphy3367. Mol Microbiol.

[28] Farkas J, Chung DW, Cha M, Copeland J, Grayeski P, Westpheling J (2013). Improved growth media and culture techniques for genetic analysis and assessment of biomass utilization by *Caldicellulosiruptor bescii*. J Ind Microbiol Biotechnol.

[29] Sambrook JaR DW (2001). Molecular Cloning: A Laboratory Manual.

[30] Kanafusa-Shinkai S, Wakayama J, Tsukamoto K, Hayashi N, Miyazaki Y, Ohmori H, Tajima K, Yokoyama H (2013). Degradation of microcrystalline cellulose and non-pretreated plant biomass by a cell-free extracellular cellulase/hemicellulase system from the extreme thermophilic bacterium *Caldicellulosiruptor bescii*. J Biosci Bioeng.

