# Supplementary material for: Deletion of Caldicellulosiruptor bescii CelA reveals its crucial role in the deconstruction of lignocellulosic biomass
Source: Biotechnol Biofuels. 2014 Oct 9;7:142. doi: 10.1186/s13068-014-0142-6 (PMC4195899; doi:10.1186/s13068-014-0142-6)
Supplement: Additional file 1: Figure S1. — Diagram of the celA (Cbes1867) deletion vector. The white colored arrows indicate sequences originating from Caldicellulosiruptor bescii and sequences originating from E. coli are indicated as black arrows. The apramycin resistant gene cassette (AprR); pSC101, low copy replication origin in E. coli; repA, a plasmid-encoded gene required for pSC101 replication; par, partition locus; pyrF cassette; 5’ and 3’ flanking sequences of the celA (Cbes1867) site in C. bescii chromosome are indicated. All Primers and the two restriction sites (KpnI and ApaLI) used in this construction are also indicated. Table S1. Primers used in this study. Table S2. Approximate Biomass Composition of insoluble substrates. [file 13068_2014_142_MOESM1_ESM.docx]

**Additional file 1**

**Additional file 1: Figure S1.** Diagram of the *celA* (Cbes1867) deletion vector**.** The white colored arrows indicate sequences originating from *Caldicellulosiruptor bescii* and sequences originating from *E. coli* are indicated as black arrows. The apramycin resistant gene cassette (Apr^R^); *pSC101,* low copy replication origin in *E. coli*; *repA*, a plasmid-encoded gene required for *pSC101* replication; *par*, partition locus; *pyrF* cassette; 5’ and 3’ flanking sequences of the *celA* (Cbes1867) site in *C. bescii* chromosome are indicated. All Primers and the two restriction sites (KpnI and ApaLI) used in this construction are also indicated.

**Additional file 1: Table S1**. Primers used in this study.

| **Primers** | **Sequences (5’ to 3’)** | **Description** |
| --- | --- | --- |
| JF006 | ATTGATTGCCAAACGCTTCATAAATCTCTAACCTC | To construct pJFW 52 |
| JF007 | TTTATGAAGCGTTTGGCAATCAATAATTAAGTAGG | To construct pJFW 52 |
| JF200 | TGTTATTTGCTATCTGTCACT | To confirm Cbes1867 (*celA*) deletion |
| DC081 | AGAGAGGTACCACCAGCCTAACTTCGATCATTGGA | To construct pJFW 52 |
| DC262 | TGTGTGGTGCACTCTGACGCTCAGTGGAACGAA | To construct pJFW 52 |
| CelA-5 | AGTGGTACCAGCGAAGATATAGCGGAGACCA | To construct pJFW 52 |
| CelA-3 | TCTTGAGTGCACCCAGCTAAAAAGTCCTATCTTG | To construct pJFW 52 |
| DC222 | TAC AAG AAA AGC CCG TCA C | Sequencing primer for pJFW 52 |
| DC228 | ATCATCCCCTTTTGCTGATG | Sequencing primer for pJFW 52 |
| DC432 | TGTTTTTACTCTCATCTTAAAACCTAAC | Sequencing primer to verify Cbes1867 deletion |
| DC435 | AACTGTACTGCATTTTAGACAA | Sequencing primer to verify Cbes1867 deletion |

**Additional file 1: Table S2**. Approximate Biomass Composition of insoluble substrates.

|  | **Glucose** | **Xylose** | **Galactose** | **Arabinose** | **Mannose** |
| --- | --- | --- | --- | --- | --- |
| *Populus trichocarpa^b^* | 49.30% | 15.80% | 2.20% | 0.85% | 2.60% |
| *Panicum virgatum* L.*^b^* | 36.80% | 19.80% | 2.90% | 3.90% | 0.56% |
| *Arabidopsis thaliana* [[1](#_ENREF_1)] | 38.90% | 20.90% | 5.20% | 4.50% | 1.00% |

*^b^*Oak Ridge National Laboratory

Unpretreated biomass, either populus or switchgrass, were analyzed for carbohydrate composition using quantitative saccharification assay NREL/TP-510-42618 and HPLC method NREL/TP-510-42623. Briefly, the samples were analyzed for carbohydrate composition using a high performance liquid chromatography (HPLC) LaChrom Elite® system (Hitachi High Technologies America, Inc.) equipped with a refractive index detector (model L-2490) and a UV–Vis detector (model L-2420). The carbohydrates glucose, xylose, galactose, mannose, and arabinose were separated using an Aminex® HPX-87P column (Bio-Rad Laboratories, Inc.), at a 0.6 mL/min flow rate of water and a column temperature of 80°C.

**Reference**

1. Van Acker R, Vanholme R, Storme V, Mortimer JC, Dupree P, Boerjan W: **Lignin biosynthesis perturbations affect secondary cell wall composition and saccharification yield in *Arabidopsis thaliana*.** *Biotechnology for biofuels* 2013, **6:**46.
